# Ethnobotanical study on wild edible plants traditionally used by Messiwa people, Morocco

**DOI:** 10.1186/s13002-022-00500-4

**Published:** 2022-03-15

**Authors:** Ridwane Ghanimi, Ahmed Ouhammou, Abdellah Ahouach, Mohamed Cherkaoui

**Affiliations:** 1 Laboratory of Pharmacology, Neurobiology, Anthropobiology, Environment, and Behaviour, Department of Biology, Faculty of Sciences Semlalia, Cadi Ayyad University, BP 2390, 40000 Marrakech, Morocco; 2 Laboratory of Microbial Biotechnologies, Agrosciences, and Environment (BioMAgE), Agrosciences, Phytobiodiversity and Environment Team, Regional Herbarium ‘MARK’, Department of Biology, Faculty of Sciences Semlalia, Cadi Ayyad University, PO Box 2390, 400001 Marrakech, Morocco

**Keywords:** Ethnobotany, Morocco, Messiwa people, Traditional knowledge, Wild edible plants

## Abstract

**Background:**

The traditional knowledge on wild edible plants has been shown in many studies a worrying decline throughout the last few decades. Therefore, the first aim of this study was to document the population knowledge on wild edible plants among the Messiwa people. The second objective was to assess the traditional knowledge of our informants according to their socio-economic status.

**Methods:**

The survey was conducted among 149 informants through a semi-structured questionnaire. The relative importance of the plants was obtained by calculating the relative frequency of citation (RFC) for each species. To compare means, we used Student's *t* test for two-group comparisons and Snedecor's *F*-test for multi-group comparisons. The multi-range Duncan test was used for multiple mean comparisons. The correspondence factor analysis (CFA) was also used.

**Results:**

A set of 64 species belonging to 56 genera from 34 families has been collected and identified. The species used for nutritional and medicinal purposes represent 56%, while 44% were used exclusively as nutritional plants. The most used parts are, respectively, the aerial parts (58%), the fruits (17%), the underground parts (13%), the seeds (8%), and finally the flowers (5%). On the other hand, the higher level of knowledge on wild edible plants was found among women, the elderly, illiterate, married people, and those engaged in agricultural occupations.

**Conclusion:**

This work could be a basis to be reproduced on other regions in Morocco and to be widened through pharmacological and nutritional studies in order to promote and valorize these wild edible plants.

## Introduction

During his history, wild edible plants (WEPs) have been an important part of the human diet [1]. Unfortunately, today, due to the development of modern agriculture and due to urbanization and globalization, the populations are becoming more and more distant from their environment [2] and the transmission of knowledge between older and younger not always assured [3, 4]. Therefore, they neglect the use of wild plants around them and the knowledge about wild edible plants is declining [5]. Indeed, according to the Yadav et al. [6], among the 300,000 plant species, 10,000 have been used for human food since the origin of agriculture, whereas this number is now only a few dozen at most. The resulting loss of agricultural and food biodiversity has become a risk to food security [7].

In Morocco, a wide variety of wild plants are used for food and healing [8–14]. Many studies on their uses have been conducted in mountain areas, mainly through ethnomedicinal surveys [15–19]. On the other hand, only a few studies have been carried out on the plains and the dietary aspect of these spontaneous plants in relation to socio-economic and cultural factors [20], whereas the wild biodiversity of the plains and the traditional knowledge of their populations on wild edible plants are very rich and threatened [21].

The first objective of this study was to document the traditional knowledge about wild edible plants (WEPs) and their uses among Messiwa people. The second aim was to evaluate the traditional knowledge of informants according to their socio-demographic and economic status. The hypothesis adopted in this study suggests that traditional knowledge increases with age, in favour of women and people with a traditional farming lifestyle.

## Methods

### Study area

**Fig. 1:**
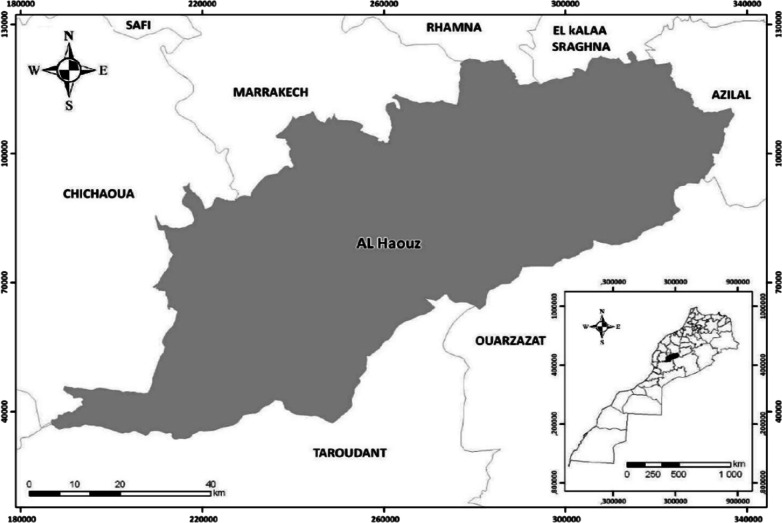
The geographical location of the study area

This study was carried out among Messiwa people who lived in a large part of Al-Haouz region, mainly in the following communes: Ait-Ourir, Ait-Faska, Ghmate, Tighedouine, Tidili-Mesfioua, Tamazouzte, Iguerferouane, Touama, and Sidi-Abdellah-Ghiat (Fig. 1). This prefecture covers 6212 km^2^, located in the south-east of Marrakech city, on the western slope of the Central High Atlas Mountains with semi-arid to sub-humid climate [22]. The topography includes plains and mountain ranges (74% of the total area) containing the highest point in Morocco (Toubkal 4167 m above sea level). The majority of the inhabitants live in rural areas (88%) with an economy based mainly on agro-pastoral activities and tourism [23]. In addition, the climate of this region ranges from humid to arid. It allows the development of a wide range of crops depending on the climate of each area [24, 25]. The first five families of the Moroccan vascular flora are *Asteraceae, Fabaceae, Poaceae, Brassicaceae*, and *Lamiaceae* [26]. The total number of species is 3913 plant species belonging to 155 families, where 640 are strict endemics [26]. On the other hand, the official language of the population of Messiwa is Tamazight, exactly the Tachelhit dialect [27]. The population of Al-Haouz is about 573,128 with an illiteracy and poverty rate of 40% and 18.3%, respectively [23]. The active population represents 57.8% (60% in the rural area and 43% in the urban area) and the most of the rural population is involved in agro-pastoral activities [23].

### Questionnaire conception

The questionnaire included two items; the first one concerns the sociocultural and economic characteristics of the informants (age, sex, civil status, monthly income, educational level, and field of work). The second item concerns the traditional knowledge of our informants on the wild edible plants (Arabic and Amazigh vernacular name, place of harvest, used part, method of use, and the reason for use).

### Data collection

The survey was conducted from January 2018 to February 2019 among Messiwa people, mostly at home, in the fields, and other workplaces. In the beginning, informed consent of the informant was obtained after having explained the content of the research. Furthermore, the interviews were conducted by Arabic and Amazigh language. Eligibility was to be of legal age, able to answer our questions, and must belong to the population of Messiwa.

Based on the questionnaires, a series of wild edible plants cited by the local population were obtained. The plant taken into consideration was the one cited by at least two informants. These plants were collected in the field with the presence of local persons. Three specimens for each plant cited were collected to ensure that the vernacular name assigned corresponded to the same biological species. Furthermore, a species may have two different vernacular names (Arabic and Amazigh) or even more. The specimens were kept under pressure in newspapers and transferred to the faculty to achieve the herbarium. A first determination was carried out in the laboratory, based on the determination manual "La flore pratique du Maroc" [28–30]. The confirmation of the determination was carried out in the regional herbarium "MARK" of the Faculty of Sciences Semlalia Marrakech-University Cadi Ayyad by one of the co-authors. Furthermore, the names of the cited species have been verified with http://www.theplantlist.org, to provide the accepted Latin names, and the specimens are present in the regional herbarium "MARK".

The relative importance of the plants was obtained by calculating the relative frequency of citation (RFC) for each species, and it was obtained by dividing the number of informants mentioning the plant by the total number of informants. The RFC value ranged from 0 to 1, with 0 indicating that no one mentioned the species and 1 indicating that all informants mentioned it [31].

The informant consensus factor was used to test the homogeneity of informants' knowledge about the use of the cited plants. The ICF was calculated using the following formula: ICF = (*Nu*_*r*_ − *N*_*t*_)/(*Nu*_*r*_ − 1), where (*Nu*_*r*_) refers to the number of use reports and (*N*_*t*_) refers to the number of taxa used. The ICF value ranged from 0 to 1, and this value indicates a high rate of consensus among informants when it tends towards 1 [32].

### Statistical analysis

All statistical analyses were performed using the statistical software SPSS,20 for Windows version 10.0.5. Descriptive statistical analysis techniques were used to test frequency and mean distributions. To compare means, we used Student's *t* test for two-group comparisons and Snedecor's *F*-test for multi-group comparisons. The multi-range Duncan test was used for multiple mean comparisons. This stepwise test compares pairs of means, controlling for comparison alpha error at a defined level.

For comparison of means, the Student’s *t* test was used to test differences between the means of two groups. In this test, we start with a null hypothesis that there is no meaningful difference between the two groups. The *t* test will prove or disprove this null hypothesis. When the number of means of groups to be compared is more than two, we used the analysis of variance (ANOVA). The *F*-test resulting from this analysis tests if whether populations’ means are equal. When a significant difference in means exists, we use the Duncan’s multiple range test (DMRT) as a post hoc test to measure specific differences between pairs of means [33].

The factorial analysis of the correspondences, noted as AFC, is an analysis technique intended for the treatment of tables of multidimensional data [34]. The main objective of this analysis is to reduce the dimension of a table with a great number of variables to another with low synthetic variables named factorial axes and noted "AFC1, AFC2, AFC3…". The AFC1 is the first factorial axis that retains the most important part of the inertia (total variance) stored in the starting space, and the AFC2 is the next axis that retains the second most important part of the inertia. The associations and oppositions existing between subjects and variables are used to measure their contribution to the total inertia for each factor. Their projection onto the factorial axes AFC1 and AFC2 enables a two-dimensional graph to be drawn, which offers aid in the interpretation of the results [35].

## Results

### The structure of studied population

Among 149 people who participated in the survey, 41 were women (27.5%) and 108 were men (72.5%). The average age of this sample was 43 years (average age of women 46 years and average age of men 42 years) and the maximum age was 82 years while the minimum was 18 years. Those under 50 years of age accounted for 71.8%, while those 50 years of age and older accounted for 28.2%. The majority of informants were married at the time of the survey (77.9%). Regarding the education level, the illiteracy rate was 38.3%; primary school level represented 24.8%, while relatively high levels represented 36.9%. Income levels were relatively low and do not exceed 3000 Moroccan Dirhams (275 euros or 335 US dollars) per month for about two-thirds of the population. Coupled with the type of profession, 43.6% of our informants worked in agriculture while 56.4% did not.

### The list of the cited species

In the beginning, 91 vernacular names were cited. The collection of the plants, in this case, showed that there are several vernacular names attributed to the same plant, also the determination of the species showed that several species have the same vernacular name. After eliminating, the plants cited only once and identifying the specimens, the final list showed 64 species belonging to 56 genera represented by 34 botanical families (Table 1). A count of 28 species was used exclusively as nutritional and the remaining 36 species were cited as both nutritional and medicinal plants.

**Table 1:** The wild edible plants consumed by Messiwa people and their ethnobotanical characteristics

Species	Family	RFC	Common name	Edible part	Food category
*Ajuga iva* (L.) Schreb	Lamiaceae	0.06	Chandgoura	Aerial part	Drink
*Allium roseum* L	Amaryllidaceae	0.02	Bsal Barri	underground part	Vegetables
*Arbutus unedo* L	Ericaceae	0.02	Sasnou	Fruits	Snacks
*Arisarum vulgare* O.Targ.Tozz	Araceae	0.04	**Irni**	underground part	Garnish
*Aristolochia paucinervis* Pomel	Aristolochiaceae	0.02	Brztm	underground part	Seasoning
*Artemisia herba-alba* Asso	Compositae	0.48	Chih	Aerial part	Drink
*Asparagus albus* L	Asparagaceae	0.19	Hmissou, **Azzou**	Aerial part (young stem)	Vegetables
*Asparagus altissimus* Munby	Asparagaceae	0.19	Hmissou, **Azzou**	Aerial part (young stem)	Vegetables
*Asparagus horridus* L	Asparagaceae	0.19	Hmissou, **Azzou**	Aerial part (young stem)	Vegetables
*Calendula arvensis* M.Bieb	Compositae	0.02	Jemra	Aerial part	Vegetables
*Capparis spinosa* L	Capparaceae	0.02	Kabbar	Fruits	Vegetables
*Caralluma europaea* (Guss.) N.E.Br	Apocynaceae	0.10	Ddaghmous	Aerial part	Drink
*Carlina gummifera* (L.) Less	Compositae	0.08	Addad	underground part, chewing gum of the flower	Vegetables
*Ceratonia siliqua* L	Leguminosae	0.19	Kharoub, **Tikida**	Fruits	Snacks
*Chamaerops humilis* L	Arecaceae	0.02	Doum	Aerial part (fruit and collar)	Snacks
*Cistus creticus* L	Cistaceae	0.02	**Irgual**	Seeds	Seasoning
*Cistus salviifolius* L	Cistaceae	0.02	**Irgual**	Seeds	Seasoning
*Cladanthus arabicus* (L.) Cass	Compositae	0.06	**Aourzid**/Tafs	Flowers	Garnish
*Cynara cardunculus* L	Compositae	0.08	Khrchouf lbaldi	Aerial part	Vegetables
*Cynodon dactylon* (L.) Pers	Poaceae	0.04	Njem, **Afar**	underground part	Seasoning
*Cyperus rotundus* L	Cyperaceae	0.02	Tamoussayt	underground part	Seasoning
*Diplotaxis* sp.	Brassicaceae	0.06	**Bahmmou**, Kerkaz	Aerial part	Vegetables
*Drimia maritima* (L.) Stearn	Asparagaceae	0.03	**Igufil**, Aansla	underground part	Vegetables
*Dysphania ambrosioides* (L.) Mosyakin&Clemants	Amaranthaceae	0.28	Mkhinza	Aerial part	Drink
*Emex spinosa* (L.) Campd	Polygonaceae	0.08	Homida	Aerial part	Vegetables
*Foeniculum vulgare* Mill	Apiaceae	0.53	Besbas	Aerial part	Garnish
*Glaucium corniculatum* (L.) Curtis	Papaveraceae	0.10	Hbbosousou/Zrriaat sarh/**Aghnbo-nouswou**	Seeds	Snacks
*Glebionis coronaria* (L.) Cass. ex Spach	Compositae	0.11	Guhouan	Flowers	Garnish
*Herniaria hirsuta* subsp. *cinerea* (DC.) Cout	Caryophyllaceae	0.16	Hrrast Lahjar	Aerial part	Drink
*Juncus acutus* L	Juncaceae	0.06	Essmar, **Azma**	Aerial part (collar, seeds)	Snacks
*Lathyrus clymenum* L	Leguminosae	0.04	**Ikikr**	Seeds	Snacks
*Lavandula dentata* L	Lamiaceae	0.04	Halhal	Aerial part	Drink
*Lavandula mairei* Humbert	Lamiaceae	0.08	**Guorzghial**	Aerial part	Drink
*Lavandula stoechas* L	Lamiaceae	0.13	Khzama	Aerial part	Drink
*Malva sylvestris* L	Malvaceae	0.53	Khobbiza, **Tibi**	Aerial part	Vegetables
*Marrubium vulgare* L	Lamiaceae	0.34	Mrouta/**Frkizoud**	Aerial part	Drink
*Mentha pulegium* L	Lamiaceae	0.40	fluo	Aerial part	Drink
*Mentha rotundifolia* (L.) Huds	Lamiaceae	0.03	Timijja Lmanta	Aerial part	Drink
*Mentha suaveolens* Ehrh	Lamiaceae	0.42	Timijja/**Timijja N'waman**	Aerial part	Drink
*Mercurialis annua* L	Euphorbiaceae	0.01	Hourrigua Lmalsa	Aerial part	Vegetables
*Morus alba* L	Moraceae	0.08	Tût, **Lmarchiq**	Fruits	Snacks
*Nasturtium officinale* R.Br	Brassicaceae	0.04	Gurnounch	Aerial part	Vegetables
*Olea oleaster* Hoffmanns. & Link	Oleaceae	0.05	Jbouj, **Azmour**	Fruits	Oil
*Ononis natrix* L	Leguminosae	0.04	**Afzdad**	Aerial part	Vegetables
*Opuntia ficus-indica* (L.) Mill	Cactaceae	0.30	Handia, **Aknari**	Fruits	Snacks
*Papaver rhoeas* L	Papaveraceae	0.08	Bellaaman/**Flilou**	Flowers	Garnish
*Peganum harmala* L	Nitrariaceae	0.11	Harmal	Seeds	Garnish
*Phoenix dactylifera* L	Arecaceae	0.08	Ablouh	Fruits	Snacks
*Portulaca oleracea* L	Portulacaceae	0.41	Trejla	Aerial part	Vegetables
*Quercus ilex* L	Fagaceae	0.03	Ballout	Fruits	Snacks
*Ridolfia segetum* (L.) Moris	Apiaceae	0.06	Tabch	Aerial part	Garnish
*Rosa canina* L	Rosaceae	0.03	**Tighfrt**	Fruits	Snacks
*Rosmarinus officinalis* L	Lamiaceae	0.33	Azir	Aerial part	Drink
*Rubia peregrina* L	Rubiaceae	0.28	Foua/**Taroubia**	underground part	Seasoning
*Rubus ulmifolius* Schott	Rosaceae	0.06	**Achddir**, **Taynajelt**	Fruits	Snacks
*Rumex pulcher* L	Polygonaceae	0.09	Selk	Aerial part	Vegetables
*Scolymus hispanicus* L	Compositae	0.28	Guernina, **Taghddiwt**	Aerial part	Vegetables
*Silene vulgaris* (Moench) Garcke	Caryophyllaceae	0.01	**Taghighacht**	Aerial part (young stem)	Vegetables
*Taraxacum getulum* Pomel	Compositae	0.02	Jemra	Aerial part	Vegetables
*Tetraclinis articulata* (Vahl) Mast	Cupressaceae	0.06	Aaraar	Aerial part	Drink
*Thymus saturejoides* Coss	Lamiaceae	0.51	Zaatar/**Azouknni**	Aerial part	Drink
*Thymus willdenowii* Boiss	Lamiaceae	0.18	Zaaitra/**Tazouknnit**	Aerial part	Drink
*Urtica dioica* L	Urticaceae	0.11	Hourrigua-lharcha	Aerial part	Drink
*Ziziphus lotus* (L.) Lam	Rhamnaceae	0.46	Nbag, **Azoguar**	Fruits	Snacks

The common names in bold are Amazigh names of plants and the other common names are in Arabic language

### Botanical families of the cited species

**Fig. 2:**
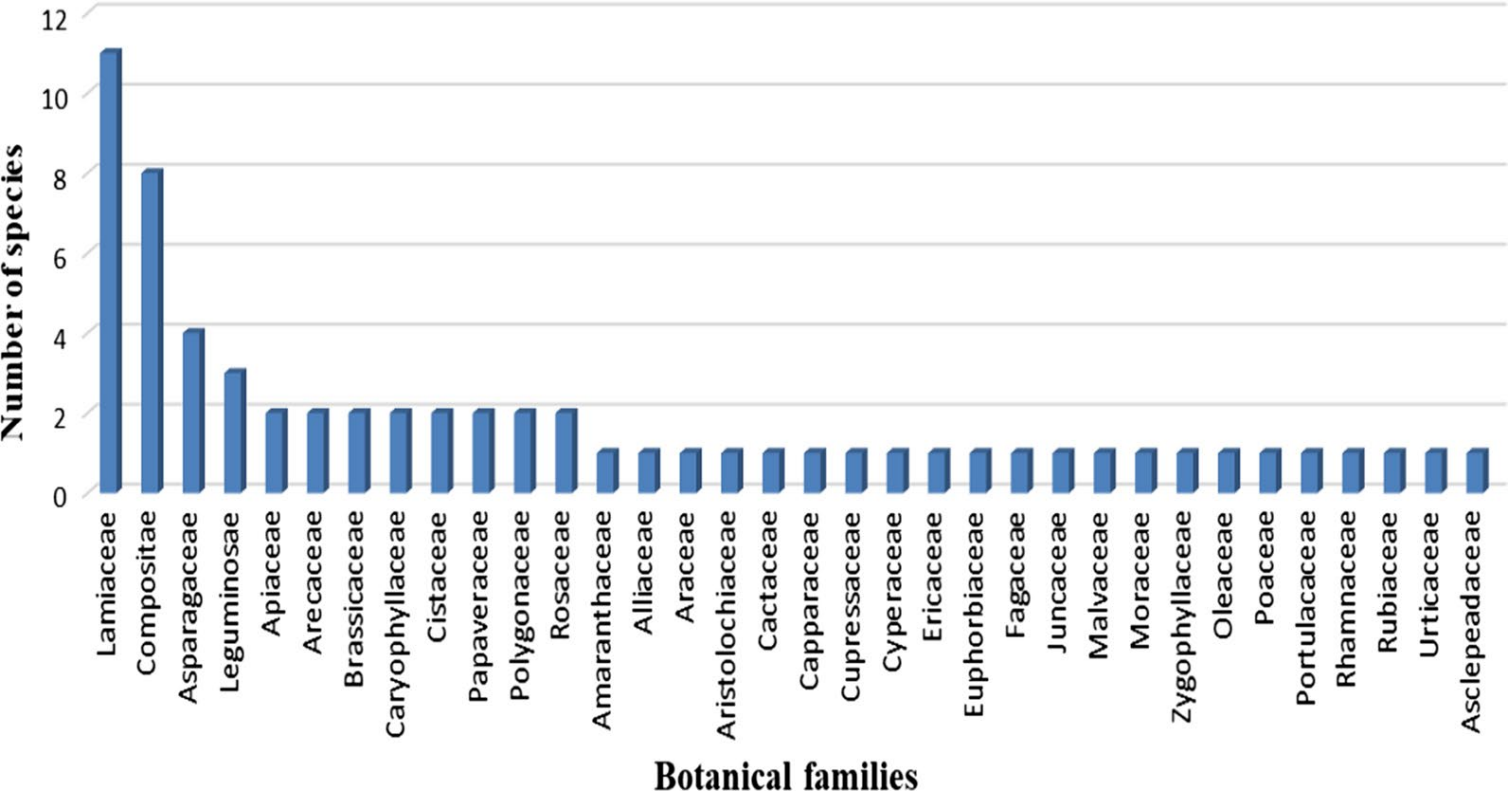
The number of species per botanical families

Figure 2 shows the number of wild edible species per families cited by Messiwa people. The *Lamiaceae* family was the most represented (Fig. 2) with 11 different species and the *Compositae* (*Asteraceae*) family comes second with 8 species. *Asparagaceae* was the third one with 4 species, followed by the family *Leguminosae* (3 species). The rest of the families were represented by only one or two species.

### Relative Frequency of citation

Four species among the 64 mentioned account for almost 25% of the total number of citations, and the top ten species account for half (50%). These ten most cited species were, respectively: *Foeniculum vulgare* Mill (RFC = 0.53), *Malva sylvestris* L. (RFC = 0.53), *Thymus saturejoides* Coss. (RFC = 0.51), *Artemisia herba-alba* Asso (RFC = 0.48), *Ziziphus lotus* (L.) Lam. (RFC = 0.46), *Mentha suaveolens* Ehrh. (RFC = 0.42), *Portulaca oleracea* L. (RFC = 0.41), *Mentha pulegium* L. (RFC = 0.40), *Marrubium vulgare* L. (RFC = 0.40). (RFC = 0.34), and *Rosmarinus officinalis* L. (RFC = 0.33).

### Informant consensus factor (ICF)

The consensus factor of the informants for wild edible plants is very high (0.95), which confirms the robustness of the information.

### The used parts

**Fig. 3:**
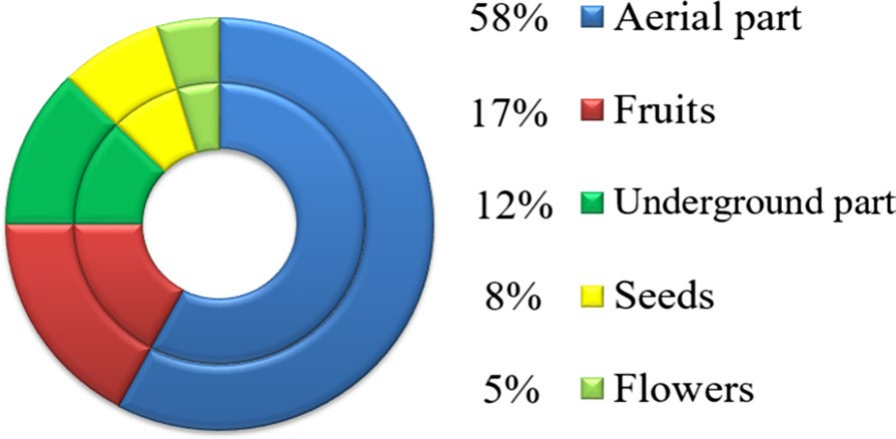
The consumed parts of the wild edible plants

Concerning the used parts of the cited WEPs (Fig. 3), the aerial part was the most used part with 37 species representing 57.8% (Fig. 3). Plants consumed for their fruits come second with a percentage of 17.2% and the majority of these plants are trees, third plants used for their underground part (12.5%), then plants used for their seeds and finally plants used for their flowers.

### The culinary uses

**Fig. 4:**
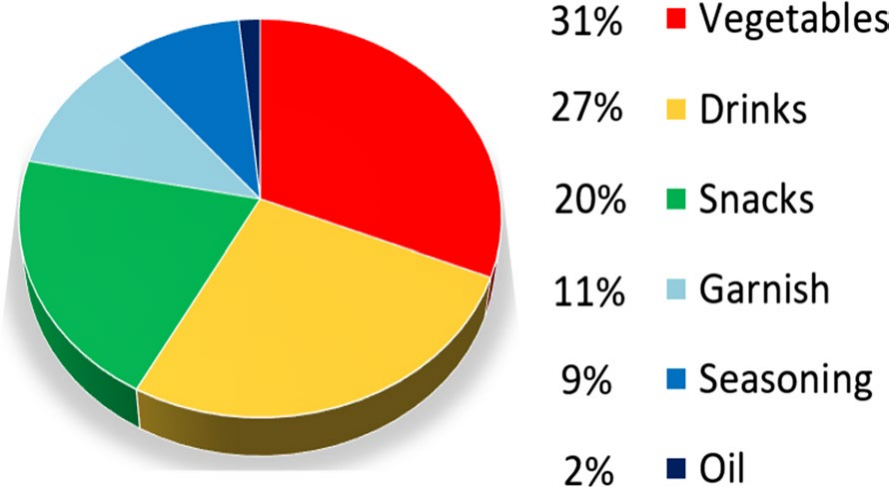
The consumption mode of wild edible plants

Based on the culinary uses and consumption patterns of wild edible plants (WEPs) cited by the population, these plants could be classified into six food categories (Fig. 4). The majority of WEPs were used as vegetables (31%), beverages account for 27%, and snacks account for 20%, of which shepherds and young people consume them raw in the fields. Other wild plants were used either as a garnish to decorate dishes (11%) or as seasoning (spices) (9%) while Olea *oleaster*, Hoffmanns. & Link., was used essentially for its oil (2%).

### Comparison of the means

A comparison of the means was carried out on the number of plants cited by our informants according to their socio-demographic and economic status (Table 2). Our results show that women have a higher level of knowledge than men do. The Student’s t test indicates that people over 50 years old have a very high level of knowledge compared to people under 50 years old. Regarding the school level, the illiterate people know an average of 11 plants; primary levels 9 plants and informants from secondary to higher education have shown only 7 plants as average. Moreover, single persons have known less than those who were married, and those who had an agricultural occupation have known much more wild edible plants than others did. Furthermore, people with a monthly income of less than 3000 *MDh* (Moroccan Dirhams) have more knowledge compared to those with more than 3000 *MDh*.

**Table 2:** Comparison of population means of cited plants according to their socio-demographic and economic status

Variables	Number	Average of plants cited	Statistical test	Homogeneous groups
Sex				
Men	108	8.6 ± 5.2	t = − 2.52 *	
Women	41	12.2 ± 8.6		
Age classes				
< 50 years	107	7.9 ± 4.4	*t* = − 4.11 ***	
50 years and over	42	13.7 ± 8.7		
School-level				
Illiterate	57	11.2 ± 7.4	*F* = 4.27 *	(1.2) (2.3)
Primary study	37	9.8 ± 5.9		
College and high school	55	7.7 ± 5.3		
Profession type				
Agriculture	65	12 ± 7.7	*t* = 4.07 ***	
Non-agriculture	84	8 ± 4.5		
Family status				
Single	33	7.2 ± 3.4	*t* = − 3.55 ***	
Already married	116	10.3 ± 7.0		
Family income				
Less than 3000 *MDh*	92	10.7 ± 7.5	*t* = 3.12*	
3000 *MDh* and more	57	7.8 ± 3.8		

t—Student’s test of comparison of 2 means; F—Fisher’s test of analysis of variance

*Test significant at the 5% level, *ns* not significant, in brackets means that the means are equal

### Correspondence factor analysis

**Fig. 5:**
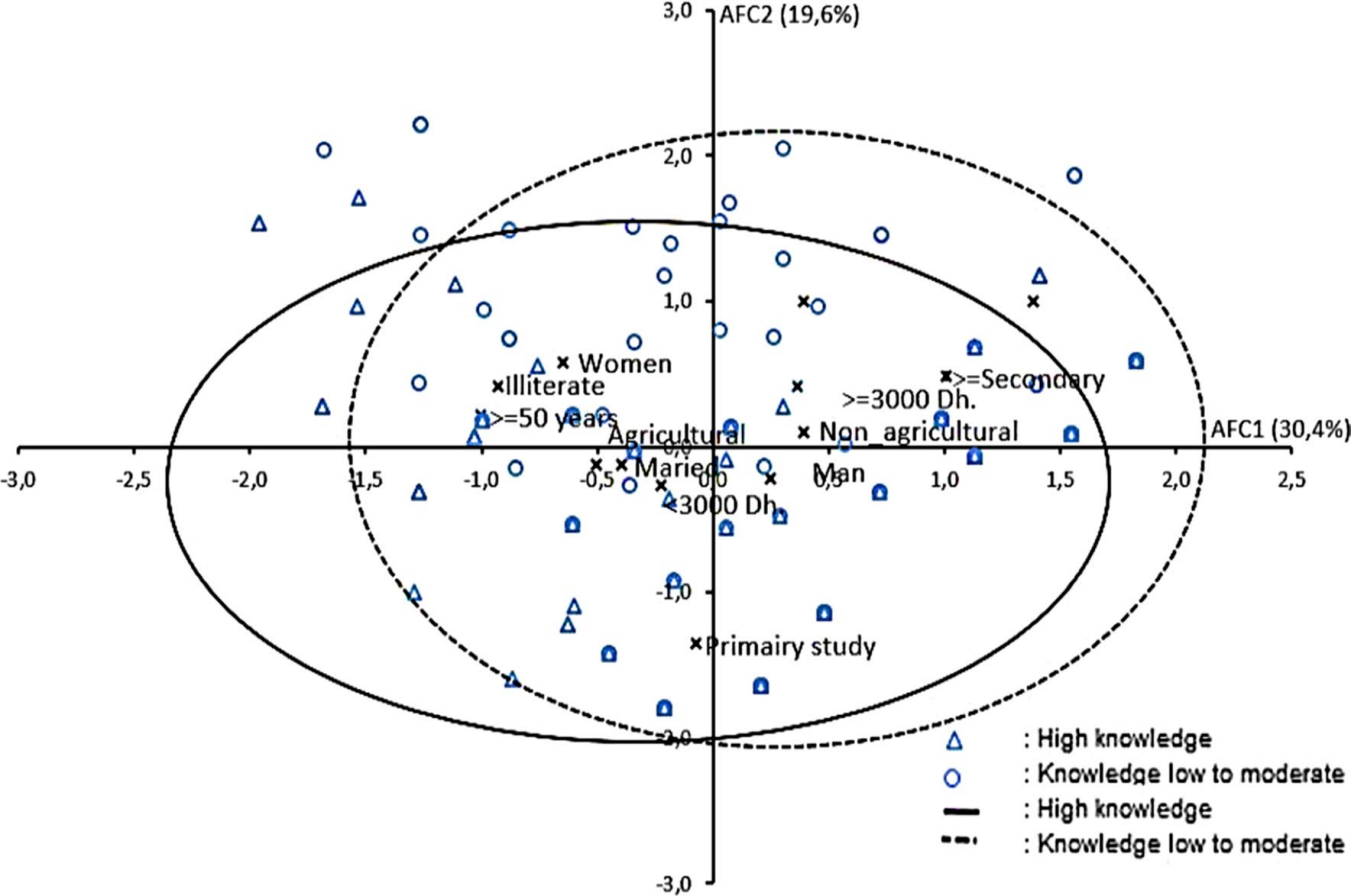
Projection in the plane 1 and 2 of the correspondence factor analysis

A correspondence factor analysis (CFA) was performed to combine socio-demographic and economic data (Fig. 5). In decreasing order, axis 1 represented 30.4% of the total inertia and expressed 70.3% of the variability for education level, 54.2% for civil status, and 39.6% for age groups. The variability for the profession, sex, and family income was very low, with, respectively, 19.9%, 16.0%, 8.1%, and 4.8% of the expressed inertia.

Based on the first axis, we could classify the households studied in two distinct groups: the most advantaged households on the negative side of the axis and the least advantaged households on the positive side. The individuals projected on the positive side were young (less than 50 years old), single, with a high school education or more, not engaged in agricultural activity, and having high incomes (more than 3000 *MDh*). In contrast, the negative side of the axis presented the following profiles: people who were aged 50 years and over, married, illiterate or with a primary school level, with agricultural activity and low income (less than 3000 *MDh*). Axis 2 accounted for 19.6% of the total variability and significantly expressed only the variability of school level (60.2%) and place of birth (45.0%).

By projecting individuals according to their level of knowledge at the factorial pattern, we observed that the ellipse of individuals with low knowledge tends towards the positive side of the factorial plane. While the ellipse of people with high knowledge tends towards the negative side. This could be interpreted as a relationship between high knowledge levels and subsistence farming lifestyle.

## Discussion

### Description of the ethnobotanical data

The first three families of the Moroccan vascular flora (*Asteraceae, Fabaceae*, and *Poaceae*) do not coincide in the same order with the most cited families in the survey (*Lamiaceae, Asteraceae*, and *Asparagaceae*). Similar study in the El-Jadida region [21] showed that the families *Asteraceae*, *Apiaceae*, and *Brassicaceae* were the richest in wild edible plants. Indeed, the two study areas in El-Jadida and Messiwa territory present different climatic conditions, which could well explain the differences in plant availability and consequently their frequency of consumption. It is accepted that the availability and abundance of wild plants in the environment of the population is related to their use although it is not the only reason [36]. In Messiwa territory, we noted in all the informants who welcomed us into their homes, consumption of beverages flavoured with species of the *Lamiaceae* family, which could explain their high RFC. They flavoured their teas with *Thymus saturejoides* Coss. "Zaatar", *Mentha suaveolens* Ehrh. "Timijja n'waman", or *Mentha pulegium* L. "Fluo" and they flavoured their coffees with *Rosmarinus officinalis* L. "Azir".

*Foeniculum vulgare* Mill, commonly known as "Besbas", was the most cited species (Table 1), highly appreciated for its flavour and its ancestrally known digestive properties [8]. It was used in many Moroccan dishes. The leaves and tender stems were used as vegetables in "Couscous" and its fruits were used to garnish and flavour bread and cakes. The ethnobotanical study conducted in Spain by Tardío et al. [37] revealed that *Foeniculum vulgare* Mill was also the most cited species. In Tbatou's study (2016) in El Jadida, it was cited second after *Lavatera cretica* L. and this may show its interest [38].

*Malva sylvestris* L., rich in vitamins and good for the stomach [39], comes in second place in our study. The same result was reported in Turkey by Dogan et al. [40]. This species was known by Messiwa people as "Tibi", "Khobbiza", or "Beqoula", and it is a famous traditional Moroccan dish cooked with spices. Informants stated that they usually consume *Malva sylvestris* L., "Khobbiza" and *Portulaca oleracea* L., "Trejla" in alternation according to availability. These two species were used in the same way to prepare the dish "khobbiza". The first species was eaten during the so-called common cold period "Lberd" (winter and spring), while the second is eaten during the summer.

In general, studies carried out on wild edible plants in Mediterranean countries had shown that many species, such as *Scolymus hispanicus* "Guernina" and *Taraxacum* sp. "Jemra", were commonly consumed, although the difference was in recipes [41].

Informants have reported on several occasions that certain species have characterized scarcity periods, for example *Scolymus hispanicus* L. "Guernina", which was used at its juvenile stage, where the leaves still tender and less thorny. Also, two species *Arisarum vulgare* O.Targ.Tozz. "Irni" and *Carlina gummifera* (L.) Less. "Addad" were eaten despite their toxicity, which resulted in several deaths according to the population's testimony. The preparation of these two species requires special handling; the method described by the informants mentions that *Arisarum vulgare* O.Targ.Tozz. must be well dried before use in order to mitigate its toxicity. When, the underground part of *Carlina gummifera* (L.) Less. must be boiled at least seven times before consumption.

Regarding young people, they have known and consumed some species directly as snacks, such as *Ziziphus lotus* (L.) Lam. "nbag", or "Tikaiine n'ouzoguar", which are widely found in uncultivated fields, while cultivated fields shelter at harvest time *Glaucium corniculatum* (L.) Curtis, which was a species also consumed as snacks by young or shepherds. This species was known among the young by the name "Hbbosousou" and among the adults by "Zrriaat-sarh" which means in the vernacular Arabic language; the shepherd's seed, also called in vernacular Amazigh language; "Aghnbo-nouswou" which means the stork's neck; because the fruit of this species is, a long pod filled with small seeds. Untended farmland and the borders of small streams have shown the presence of certain species used as vegetables, such as *Rumex pulcher* L. "Selk" and *Emex spinosa* (L.) Campd. "Hommida".

The majority of the wild edible plants in this study were used for their aerial parts, which agrees with the results of several authors [38, 42–44]. Some species, such as *Opuntia ficus-indica* (L.) Mill, *Ceratonia siliqua* L., *Quercus ilex* L., and *Arbutus unedo* L., were consumed for their fruits. These species were commonly consumed in several countries of the Mediterranean basin [40, 41, 45]. This similarity was governed by the common characteristics of the Mediterranean climate [40], although differences can be seen in the traditional recipes for each studied region [41].

Wild edible plants had several modes of consumption and preparation methods in different traditional recipes [40]. The large part of these WEPs were eaten cooked [40, 42], which can be explained by their use as vegetables.

During scarcity periods and up to the present time, the Messiwa people have consumed the following species: *Malva sylvestris* L. "Khobbiza", *Portulaca oleracea* L. "Trejla", and *Scolymus hispanicus* L. "Guernina". The first two species were cooked alone to prepare the dish of “Khobbiza”, whereas *Scolymus hispanicus* L. "Guernina" was cooked in other traditional recipes. Besides, *Caralluma europaea* (Guss.) N.E.Br. was eaten recently raw with milk for diabetics or with honey for people with cancer. Thus, for the rest of the species, several methods of preparation exist.

### The population's knowledge

According to our results men have a low level of knowledge compared to women, which agrees with several studies such as the study of Tbatou et al. [38] in El-Jadida region. Other studies have shown the opposite, as in some Latin American and West African countries where most wild edible fruits are consumed by men when they are in the bush to prepare the fields or when they hunt [46, 47]. In addition, since age is naturally associated with the learning process and time helps individuals to accumulate knowledge and experiences, the older people showed great knowledge compared to the younger ones [48], whereas this knowledge could be inversely proportional to the level of education, as the case in this paper [49, 50]. Moreover, the positive correlation between high knowledge and the variables marriage and agricultural activity may be due to how married people have more responsibility for ensuring the household's subsistence and money needs [51].

## Conclusion

As the traditional knowledge of wild edible plants and the plant biodiversity suffers from continuous erosion, the ethnobotanical studies are required to document this heritage in order to preserve and promote these species. In this study, a set of 64 species were cited as wild edible plants. Our informants also cited the preparation methods and the medicinal uses of these plants. The most used parts are, respectively, the aerial parts (58%), the fruits (17%), the underground parts (13%), the seeds (8%), and finally the flowers (5%). On the other hand, the higher level of knowledge about these wild edible plants was found among women, the elderly, illiterate, married people, and those engaged in agricultural occupations. This work could be a basis to be reproduced on other regions in Morocco and to be widened through pharmacological and nutritional studies in order to promote and valorize these wild species.

## Data Availability

The datasets used and/or analysed during the current study and the questionnaire are available from the corresponding author on reasonable request.

## Appendix 1

See Table 3.

**Table 3:** The list of plants cited by a single informant

Species	Family	RFC	Common name	Edible part	Food category
*Anagyris foetida* L	Fabaceae	0.007	Fûl l-kalb	Fruits	Snacks
*Cupressus dupreziana* var. atlantica (Gaussen) Silba	Cupressaceae	0.007	Sawr al-atlas	Fruits	Drink
*Euphorbia resinifera* O.Berg	Euphorbiaceae	0.007	Zaqqûm	Aerial part	Drink
*Juniperus thurifera* var. africana Maire	Cupressaceae	0.007	Adruman	Fruits	Drink
*Pistacia atlantica* Desf	Anacardiaceae	0.007	Labtem	Aerial part (fruits and leafs)	Drink
*Pistacia lentiscus* L	Anacardiaceae	0.007	Dro	Aerial part (fruits and leafs)	Drink
*Populus nigra* L	Salicaceae	0.007	Safsaf	Aerial part (buds and leafs)	Drink
*Rosa centifolia* L	Rosaceae	0.007	Lwerd	Flowers	Drink

## Appendix 2

See Table 4.

**Table 4:** The list of plants mentioned as both nutritional and medicinal species

Species	Family	RFC	Common name	Form of preparation
*Tetraclinis articulata* (Vahl) Mast	Cupressaceae	0.06	Aaraar	Aerial part as drink
*Ononis natrix* L	Leguminosae	0.04	**Afzdad**	Aerial part as vegetables
*Cladanthus arabicus* (L.) Cass	Compositae	0.06	**Aourzid**/Tafs	Flowers
*Rosmarinus officinalis* L	Lamiaceae	0.33	Azir	Aerial part
*Papaver rhoeas* L	Papaveraceae	0.08	Bellaaman/**Flilou**	Flowers
*Foeniculum vulgare* Mill	Apiaceae	0.53	Besbas	Aerial part
*Ajuga iva* (L.) Schreb	Lamiaceae	0.06	Chandgoura	Aerial part
*Artemisia herba-alba* Asso	Compositae	0.48	Chih	Aerial part
*Caralluma europaea* (Guss.) N.E.Br	Apocynaceae	0.1	Ddaghmous	Aerial part
*Mentha pulegium* L	Lamiaceae	0.4	fluo	Aerial part
*Rubia peregrina* L	Rubiaceae	0.28	Foua/**Taroubia**	Underground part
*Lavandula mairei* Humbert	Lamiaceae	0.08	**Guorzghial**	Aerial part
*Nasturtium officinale* R.Br	Brassicaceae	0.04	Gurnounch	Aerial part
*Lavandula dentata* L	Lamiaceae	0.04	Halhal	Aerial part
*Peganum harmala* L	Nitrariaceae	0.11	Harmal	Seeds
*Asparagus albus* L	Asparagaceae	0.19	Hmissou, **Azzou**	Aerial part (young stem)
*Asparagus altissimus* Munby	Asparagaceae	0.19	Hmissou, **Azzou**	Aerial part (young stem)
*Asparagus horridus* L	Asparagaceae	0.19	Hmissou, **Azzou**	Aerial part (young stem)
*Mercurialis annua* L	Euphorbiaceae	0.01	Hourrigua Lmalsa	Aerial part
*Urtica dioica* L	Urticaceae	0.11	Hourrigua-lharcha	Aerial part
*Herniaria hirsuta* subsp. *cinerea* (DC.) Cout	Caryophyllaceae	0.16	Hrrast Lahjar	Aerial part
*Cistus creticus* L	Cistaceae	0.02	**Irgual**	Seeds
*Cistus salviifolius* L	Cistaceae	0.02	**Irgual**	Seeds
*Capparis spinosa* L	Capparaceae	0.02	Kabbar	Fruits
*Ceratonia siliqua* L	Leguminosae	0.19	Kharoub, **Tikida**	Fruits
*Lavandula stoechas* L	Lamiaceae	0.13	Khzama	Aerial part
*Dysphania ambrosioides* (L.) Mosyakin&Clemants	Amaranthaceae	0.28	Mkhinza	Aerial part
*Marrubium vulgare* L	Lamiaceae	0.34	Mrouta/**Frkizoud**	Aerial part
*Ziziphus lotus* (L.) Lam	Rhamnaceae	0.46	Nbag, **Azoguar**	Fruits
*Ridolfia segetum* (L.) Moris	Apiaceae	0.06	Tabch	Aerial part
*Silene vulgaris* (Moench) Garcke	Caryophyllaceae	0.01	**Taghighacht**	Aerial part (young stem)
*Cyperus rotundus* L	Cyperaceae	0.02	Tamoussayt	Underground part
*Mentha rotundifolia* (L.) Huds	Lamiaceae	0.03	Timijja Lmanta	Aerial part
*Mentha suaveolens* Ehrh	Lamiaceae	0.42	Timijja/**Timijja N'waman**	Aerial part
*Thymus willdenowii* Boiss	Lamiaceae	0.18	Zaaitra/**Tazouknnit**	Aerial part
*Thymus saturejoides* Coss	Lamiaceae	0.51	Zaatar/**Azouknni**	Aerial part

The common names in bold are Amazigh names of plants and the other common names are in Arabic language

## Abbreviations

WEPs: Wild edible plants
RFC: Relative frequency of citation
